# Missing tissue, missing data: Resolving brain volume loss caused by anti-amyloid therapies

**DOI:** 10.1371/journal.pmed.1004880

**Published:** 2026-01-20

**Authors:** Francesca Alves, Scott Ayton

**Affiliations:** 1 The Florey Institute of Neuroscience and Mental Health, Melbourne, Australia; 2 Florey Department of Neuroscience and Mental Health, The University of Melbourne, Melbourne, Australia

## Abstract

Anti-amyloid drugs modestly slow Alzheimer’s disease progression, albeit with uncertainty of sustained benefit, particularly as they cause paradoxical acceleration of brain volume changes. Here, we examine explanations for these volume changes and argue for transparent release of clinical trial data.

Alzheimer’s disease (AD) is defined by amyloid-β (Aβ) plaque accumulation, progressive neurodegeneration, brain volume loss, as observed by magnetic resonance imaging (MRI), and concomitant neurocognitive decline. Coordinated efforts across the entire sector, including patients, researchers, clinicians, and industry partners, have been instrumental in establishing Aβ as a therapeutic target and the subsequent development of landmark anti-Aβ treatments for AD. The approved agents, lecanemab and donanemab, slow cognitive deterioration, although showed modest effect sizes in clinical trials lasting 18 months [1,2], and amyloid-related imaging abnormalities (ARIA) were a common side effect. ARIA are MRI-detected side effects of anti-Aβ therapies and typically present as brain swelling (ARIA-E) or small areas of bleeding (ARIA-H). While often asymptomatic, ARIA can cause headache, confusion, visual disturbances, or focal neurological symptoms in some individuals. The long-term impact of ARIA is unknown, especially when ARIA is more severe.

While we await evidence from long-term trials, MRI-detectable brain volume changes, reflecting neurodegeneration, offer a surrogate indicator of AD progression. Paradoxically, anti-Αβ therapies cause accelerated volumetric change, particularly in people who develop ARIA (Fig 1) [3].

**Fig 1:**
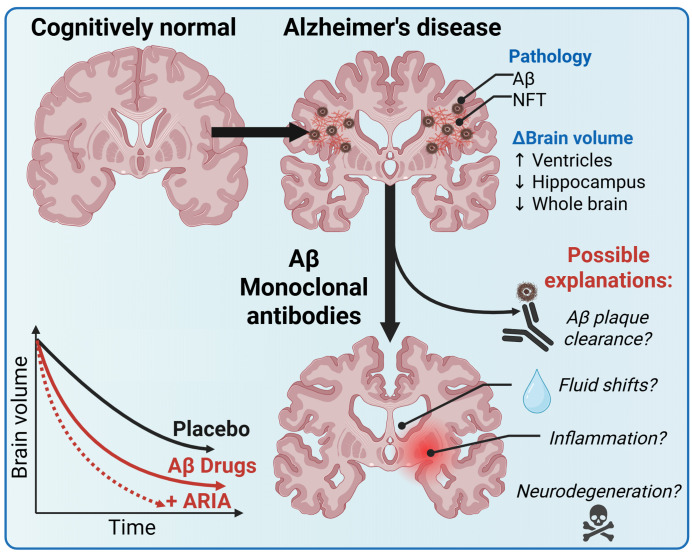
Accelerated brain volume loss with anti-amyloid monoclonal antibodies and proposed mechanisms. Clinical progression of Alzheimer’s disease is characterized by brain volume loss (enlargement of the ventricles with atrophy of the hippocampus and whole brain). Anti-amyloid monoclonal antibodies exacerbate brain volume loss, particularly in patients that experienced amyloid-related imaging abnormality (ARIA). Plausible explanations for volume loss include removal of amyloid, fluid shifts, inflammation, or neurodegeneration. Figure created using BioRender: Ayton, S. (2025) https://BioRender.com/hymirt9.

The reason for accelerated volume change caused by these drugs is uncertain, warranting concern. Clinical outcomes hold priority over surrogate outcomes such as volumetrics, but when the clinical importance of the effect is uncertain, volume changes may provide perspective. There has been considerable interest in these results [4–7], but there has been limited release of these data by the study sponsors, causing uncertainty for patients, treating physicians and researchers.

Filling this void, a series of explanations have been proposed (Fig 1). First, it was proposed that the clearing of Αβ plaque and the volume it occupies may account for the volume change. However, even using generous estimates, the dry weight of Αβ can only account for <1/1,000 of the volume change caused by these drugs [4]. It has been argued that plaque loss may account for this change, since plaque is composed of more than just Αβ [7]. However, the non-Αβ components of plaque would need to occupy three orders of magnitude more volume than Αβ does to account for this degree of change. Recently, a post-mortem study showed that anti-Αβ therapies reduce plaque in only the superficial layers (cortical layer 1) of the cortex, further restricting the possible volume of plaque that could contribute to the overall volume change [8]. In addition, there is a temporal shift in loss of plaque detectable by Positron Emission Tomography scanning that occurs ~6 months prior to detectable volumetric change, which is not easily reconcilable with a hypothesis that plaque volume change accounts for MRI volume change [3]. There is also a spatial disconnect, with volume loss occurring in areas with little plaque, such as white matter [3]. Therefore, the evidence that Aβ or plaque removal could plausibly account for volume change is challenged by quantitative, spatial, and temporal discrepancies.

Resolution of inflammation has also been proposed as a mechanism underlying treatment-related volume change. However, ARIA itself reflects an MRI-detectable inflammatory reaction. If dampening inflammation increased brain volume loss, it is paradoxical that ARIA, an inflammatory response, also correlates with greater volumetric change [3]. Moreover, recent high-resolution spatial transcriptomics and single-cell RNA-sequencing of post-mortem cases treated with anti-Αβ therapy revealed increased inflammation, characterized by activation of the immune complement system and upregulation of disease-associated microglial markers, apolipoprotein E (APOE), and triggering receptor expressed on myeloid cells 2 [9]. These findings offer a mechanistic basis by which inflammation triggered by Αβ clearance could inadvertently drive neuronal loss, providing a plausible off-target pathway linking Αβ removal to neurodegeneration. In support of this possibility, the only paper to have investigated neuronal counts after anti-Αβ therapy revealed increased neuronal loss - the definition of neurodegeneration [10].

While clinical outcomes rightfully are prioritized over surrogate outcomes, volumetric changes are safety signals where conservative interpretations should be applied. In AD research, volumetric changes are interpreted as neurodegeneration, and it is right to adhere to the conventional interpretation unless data directs us otherwise. Offering alternative explanations that are inconsistent with the data risks obscuring genuine biological insights. If, using our conventional understanding, volumetric change indeed reflects iatrogenic neurodegeneration, this does not preclude a net benefit of the drugs. It is possible that treatment selectively eliminates dysfunctional neurons that contribute to pathology, or that Aβ removal produces some collateral cellular damage. If the latter, this may explain why longer-term benefits are not sustained for anti-Aβ therapies, but it also reveals a modifiable mechanism and an opportunity to refine them to preserve their benefits while minimizing harm. But we lack the data to make definitive interpretations.

Much of our uncertainty about the meaning of the observed volume changes is unnecessary. The clinical trials have already gathered data that could offer decisive insight. For example, volumetric changes could be examined after stratifying participants by ARIA status and APOE genotype (the top genetic risk factor for AD), by analyzing the temporal and regional relationship between volume loss and plaque reduction (particularly areas not associated with plaque), and by evaluating how volumetric changes relate to clinical outcomes. Such analyses would substantially clarify the underlying biology.

That is why we are renewing our call for pharmaceutical companies that own these data to present these transparently. More than 3 years since the lecanemab clinical trial was published [1], the article mentioned that the MRI volumetric data have not yet been analyzed, but we still await the publication of these findings. Preliminary volumetric analysis for donanemab has been published for the phase III trial [11], but volumetric change in different regions, with stratifications for ARIA and associations with clinical outcomes have not been reported.

The successful development of transformative therapeutics requires coordinated engagement across the biomedical ecosystem, uniting basic scientists, clinicians, and industry. The Transparency and Openness Promotion guidelines (first released in 2015 [12], recently updated in 2025 [13]) were designed to enhance the verifiability of empirical research by promoting replication, preregistration, transparent reporting, and the availability of materials, data, and code. Major journals now enforce these standards, recognizing that open data and rigorous documentation are essential for credibility and scientific progress. In this regard, academic researchers play a critical role in independently interpreting such data by scrutinizing results without the commercial pressures that can influence analyses.

Yet this cultural shift toward transparency has apparently not extended to the pharmaceutical sector in this case. This is notable given that pharmaceutical companies: (1) conduct large-scale clinical trials built on foundational discoveries generated within open academic science, for which they rightfully profit; and (2) expose human participants to investigational therapies and associated risks, and the data regarding those risks are held solely with the sponsor. It is therefore reasonable to expect industry sponsors, in particular, to embrace data transparency. Doing so would directly inform how these therapies are delivered in practice, accelerate our understanding of their safety profiles, and help identify which patients are most likely to benefit.

Whether the observed volumetric changes represent a true safety signal, a benign physiological response, or even a marker of efficacy remains unresolved. Meaningful scientific debate will remain constrained as long as key clinical trial datasets are inaccessible, limiting the guidance we can provide to patients and clinicians. As central contributors to biomedical progress and beneficiaries of public trust, pharmaceutical companies carry a responsibility to provide the same level of transparency that the broader research community upholds.

## Abbreviations

Aβ: amyloid-β
AD: Alzheimer’s disease
APOE: apolipoprotein E
ARIA: amyloid-related imaging abnormalities
MRI: magnetic resonance imaging
